# Attention to Emotional Information Is Associated With Cytokine Responses to Psychological Stress

**DOI:** 10.3389/fnins.2018.00687

**Published:** 2018-10-02

**Authors:** Viktoriya Maydych, Maren Claus, Carsten Watzl, Thomas Kleinsorge

**Affiliations:** ^1^Department Psychology and Neurosciences, Leibniz Research Centre for Working Environment and Human Factors, TU Dortmund (IfADo), Dortmund, Germany; ^2^Department Immunology, Leibniz Research Centre for Working Environment and Human Factors, TU Dortmund (IfADo), Dortmund, Germany

**Keywords:** inflammation, cytokines, saliva, psychological stress, attention networks, emotional information

## Abstract

This study aimed to investigate the impact of mental stress on salivary cytokines and attention to emotional stimuli, as well as associations between stress-induced changes of immune and cognitive parameters. In a randomized order a total of 60 young adults were assigned to one of two stress conditions with varying stress intensity. High stress was induced by a socially evaluated Paced Auditory Serial Addition Test (PASAT). As a low stress task a paper-and-pencil version of PASAT was administered. Salivary cytokines were measured before, 5 min after, and 45 min after completion of the stress task, and were assayed for pro- and anti-inflammatory cytokines. Three distinct types of attention – alerting, orienting, and executive control – were measured by the modified Emotional Attention Network Test Integration (E-ANTI). IL-1β and IL-6 increased only in the high-stress group. Significant increases in IFN-α, IFN-γ, TNF-α, and IL-10 at 45 min after stress induction (all *p*’s < 0.05) were observed in both the high-stress and the low-stress group. Alerting attention was positively related to more pronounced increases in IFN-α and TNF-α in both groups. Further, better orienting attention after presentation of negative cues was associated with higher increases in IFN-α, TNF-α, IL-2, IL-5, and IL-10 in both groups, and higher overall levels of IFN-α, IFN-γ, and IL-12p70 in the high-stress group. There were no systematic gender differences in cytokine responses. We conclude that attention processes modulate the increases of salivary cytokines after stress exposure, and that these effects depend on stress level, particular attention network, and stimulus valence.

## Introduction

Considerable evidence indicates that psychological stress can lead to alterations of the immune system (Segerstrom and Miller, 2004). Apart from studies on changes of immune cell counts, proportions and functions in response to stress, recent research has focused on the question how stress affects markers of inflammation. Previous studies demonstrated that acute psychological stress is associated with an increase of pro-inflammatory cytokines, such as IL-1β, IL-6, and tumor necrosis factor TNF-α (Steptoe et al., 2007; Marsland et al., 2017). These inflammatory markers are in turn suggested to be involved in a variety of diseases which implicate inflammation. Along with physical diseases, e.g., atherosclerosis (Moss and Ramji, 2016), autoimmune disease (Brennan and Feldmann, 1996), or cancer (Dranoff, 2004), growing evidence associates cytokines with psychopathology, e.g., depression (Miller et al., 2009) and anxiety (Hou et al., 2017). However, the results reported so far are heterogeneous with respect to different biomarkers and variable time points of sampling. In addition, due to small sample sizes little is known about potential moderators that may alter cytokine stress responses (for review see Slavish et al., 2015; Marsland et al., 2017).

One factor hypothesized to be associated with cytokine stress responses is attention. As a major pathway of emotion regulation, attention processes are obviously implicated in stress responses. For example, by attentional engagement an individual can focus on potentially threatening aspects of the stressful event, thus intensifying the experience of stress, or ignore them and thereby possibly dampen the stress response (Gross, 2001; Compton et al., 2013). According to the framework suggested by Posner and Petersen (1990), the attention system can be subdivided into three functionally and anatomically separate subsystems: alerting, orienting, and executive control. The alerting system subsumes tonic alertness (i.e., sustained activation of the cognitive system over a period of time) and phasic alertness (i.e., increased response readiness when a warning signal is presented prior to a target stimulus). Orienting involves the selection of specific information from sensory input. Finally, executive control is an effortful mental process which is engaged during monitoring and resolving conflict between responses (Fan and Posner, 2004). A few studies suggested that alerting and orienting attention declined after anxiety induction (Pacheco-Unguetti et al., 2010; Garner et al., 2012). In contrast, executive control was not compromised by stress. Further support for selective effects of stress on attention networks comes from a controlled laboratory study with young children who underwent the Trier Social Stress Test for children (Fairbairn, 2007). The author found detrimental effects of stress on orienting network, but only in males. In addition, higher cortisol levels tended to be positively related to better orienting. Taken together, previous research demonstrates that stress affects alerting and orienting network, and these effects are associated with physiological stress responses.

To date, there is little evidence on the relationship between cytokine stress responses and attention processes. One recent attempt to fill this gap is a study by Newton et al. (2017). These authors investigated the effects of two different laboratory stressors (Trier Social Stress Test, Angry Memory Retrieval) on cytokine reactivity while varying the opportunity for post-stressor rumination. The authors found some evidence that an increase of salivary IL-1β in the condition with reduced rumination was less pronounced as compared to a rest condition. This finding is consistent with clinical literature linking altered attention processes with poorer health outcomes, e.g., depression (Mathews, 1990; Mogg and Bradley, 2005; Williams et al., 2009). Considering that inflammation is implicated in a broad variety of stress-related diseases (e.g., Aschbacher et al., 2012), the study of potential psychological mediators of cytokine reactivity to acute stress may pave the way toward interventions that protect against the development of severe health consequences following stress.

In the current study, we varied the intensity of a laboratory mental stressor between two groups of participants, before and after which we assessed levels of saliva cytokines. As recommended by Slavish et al. (2015), both pro- and anti-inflammatory cytokines were assayed. The intensity of laboratory mental stress (PASAT) was manipulated by including socially evaluative stress and increasing difficulty levels from trial to trial in the high-stress condition, whereas the low-stress condition consisted of the equivalent paper-pencil arithmetic task. Task difficulty was kept constant and social evaluative stress was lacking in this condition. In addition, we examined stress effects on the efficiency of attention networks measured by an affective variant of the Attention Network Test (ANT, Fan et al., 2002). Apart from separately examining how stress modulated attention networks and cytokine reactivity, we also explored eventual interrelations between both systems as a function of stress.

## Materials and Methods

### Participants

Participants were 60 healthy adults (30 men; 30 women) aged 19–35 years (*M* = 25.25, *SD* = 3.4). Study exclusion criteria were smoking, drug and/or alcohol abuse, pregnancy and/or nursing, acute and/or chronic infections, psychiatric diseases, and any sort of medication. Participants’ body mass index was normal (*M* = 23.25, *SD* = 2.6). All participants were contacted 2–3 days prior to the visit and instructed to abstain from alcohol, not to exercise and to avoid food with high fat content 1 day before and on the day of testing. In addition, we asked participants to forgo any meals for at least 1 h and taking any drinks for at least 30 min. before the experimental session. Volunteers received either 20€ or course credits for their participation. All participants signed informed consent after arrival at the laboratory.

### Procedure

Participants were tested individually. They arrived at the laboratory between 2 p.m. and 3.30 p.m. After signing the informed consent, saliva samples were taken. Saliva was sampled using the Salivette^®^ Cortisol with synthetic swab (Sarstedt). The swab was placed in the mouth of the participant for 1 min with gentle movement. The saliva-soaked swab was immediately stored on ice. Samples were centrifuged and saliva was aliquoted and stored at -80°C until analysis.

Next, participants filled out questionnaires on their actual stress and anxiety level. Each participant was then randomly assigned to one of the stress conditions. Then, they were asked to perform the corresponding version of the PASAT (cf. Stress protocol), which took about 25 min. After that, the same questionnaires on stress and anxiety level were filled out, which was followed by the second sampling of saliva. Next, participants completed the E-ANT task. Finally, a third saliva sample was drawn and participants were debriefed.

### Measures and Materials

#### Subjective Measures

Subjective stress experience was measured by means of a visual analog scale (VAS). The scale comprised a continuous horizontal line 20 centimeters in length, anchored by 2 verbal descriptors (*“not stressed at all”* and *“extremely stressed”*). The instruction consisted of the question “*How stressed do you feel in the moment?*”.

Anxiety was assessed by the state anxiety scale of the State-Trait Anxiety Inventory (STAI-S; Spielberger, 1972). The scale refers to current feelings of fear, nervousness, discomfort etc. The intensity of these feelings was rated on a four point Likert scale (1 = not at all, 4 = very much). To calculate a current anxiety score, single items ratings were summed up (after recoding items with inverse valence).

#### Cytokine Assays

Cytokine levels in saliva were measured using the LEGENDplex Human Inflammation Panel (13-plex) and the LEGENDplex Human Th1/Th2 Panel (8-plex) (both from BioLegend) according to the manufacturer’s instructions with slight modifications.

Briefly, 10 μl saliva or standard was added to a V-bottom 96 well plate and mixed with 30 μl assay buffer, 10 μl beads and 10 μl biotinylated detection antibody mix and incubated for 2 h at RT on a shaker at 600 rpm. Then, 10 μl PE-conjugated Streptavidin was added, followed by an additional incubation for 30 min at RT on a shaker at 600 rpm. After two washes with wash buffer (provided in the kit), PE fluorescence intensity of the beads was measured on a LSRFortessa flow cytometer (BD Biosciences). Beads populations were identified by Fsc/Ssc features and fluorescence intensity in the APC channel. Approx. 400 beads per analyte were acquired. Data were analyzed using the LEGENDplex^TM^ Data Analysis Software (VigeneTech). All samples were analyzed at the same day to avoid inter-assay variation. According to the manufacturer, intra-assay variation is between 3 and 16% CV.

#### Stress Protocol

In the high-stress condition, the PASAT was applied (Gronwall, 1977). The task consisted of series of numbers from 1 to 9 presented in a random order by the PC loudspeaker. Participants were asked to add each digit to the one presented prior to it and speak out the answers. We applied a PASAT version developed by Veldhuijzen van Zanten et al. (2004, 2009). These authors modified the task by including additional social evaluative stress. Participants were videotaped during task performance and their facial reactions were displayed in real time on a monitor. They were also given an instruction to keep looking at the screen and were told that two senior researchers would later evaluate the tape for body language during task performance. In our setting, the female experimenter was sitting diagonally opposite to the participant and checked his or her answers for correctness. Task difficulty was adapted in accordance with participants’ performance in the following way. Task difficulty was defined in terms of the inter-stimulus interval (ISI) at which the numbers were presented. The task consisted of a practice run and 2 blocks of 4 trials each. The trials consisted of 33 digits presented at ISIs of 3.6 s (first difficulty level), 38 digits presented at ISIs of 3.2 s (second difficulty level), 43 digits presented at ISIs of 2.8 s (third difficulty level), 50 digits presented at ISIs of 2.4 s (fourth difficulty level), and 55 digits presented at ISIs of 2 s (fifth difficulty level). For each difficulty level, 8 different versions were available. If participants completed the practice run or any of the experimental trials without error, the next but one difficulty level was presented. In case they had made more than four errors in the row, they were presented with another version of the same difficulty level.

In the low-stress condition, the PASAT was replaced by a paper-and-pencil version of the task. Participants were required to add the same series of single digits for approximately the same duration as the PASAT. During this time the experimenter sat 1 m diagonally opposite to the participant measuring time and monitoring task performance. In this condition, participants were not exposed to socially evaluative stress. No visual feedback, no instruction about alleged analyses of body language were given. Mental stress was also reduced by keeping the difficulty level constant. A low-stress condition was preferred to a basal rest condition in order to ensure that cytokine responses were elicited by variations in stress reaction and not by secondary characteristics of the experimental setting, such as, e.g., performing mental arithmetic, differences in body posture etc. (Het et al., 2009).

#### E-ANTI

To assess attention to emotional stimuli, we used the modified version of the ANT based on Cohen and colleagues (emotional attention network test integration E-ANTI, Cohen et al., 2011). This experimental task is based on a 6-factorial within-participants design comprising the factors Tone (tone or no tone), Cue Valence (positive or negative), Cue Validity (valid or invalid), Target Congruity (congruent or incongruent), Target Valence (positive or negative), and Cue-Target Congruity (congruent or incongruent). Participants’ task was to detect the valence of a central stimulus (target) comprising a smiley (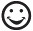) or frowney (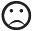) that was flanked by four other smileys or frowneys (two per side). The flanker stimuli were either identical to the target (e.g., 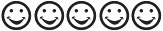), resulting in a target congruent condition, or from the opposite category (e.g., 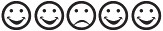), resulting in a target incongruent condition. These stimuli were presented in the upper or lower half of the computer screen. The presentation of target plus flankers was preceded by the following sequence of events. In 50% of the trials, an alerting signal (auditory tone) was presented at the start of a trial. In the other 50% of trials, the tone was absent. Afterward, a cue (i.e., a positively or negatively valenced emotional picture) appeared in the upper or lower half of the screen. When cue and target were presented at the same position, the cue was valid, otherwise it was invalid. Furthermore, as both the cue and the target could be positively or negatively valenced, the valence of these stimuli was either congruent (e.g., a smiley preceded by a positively valenced picture) or incongruent (e.g., a smiley presented by a negatively valenced picture), constituting the factor Cue-Target Congruity. The number of combinations of the factors was balanced and presented in a random order. The task comprised 14 blocks of 64 trials each.

The alerting stimulus was a 84 dB (2000 Hz) tone. Orienting cues were 10 positive and 10 negative pictures (5.42 cm × 4.06 cm) selected from International Affective Picture System (IAPS, Bradley et al., 2001). The picture selection was based on valence and arousal ratings given by additional sample of participants. The selection procedure is described in Kleinsorge (2009) in detail. The selected pictures were low-arousing (*M* = 1.64, *SD* = 0.19) positive (*M* = 3.97, *SD* = 0.43), and low arousing (*M* = 1.82, *SD* = 0.21) negative (*M* = 1.90, *SD* = 0.24) pictures. The target/distractor stimuli were rows of five positive and/or negative emoticons.

**FIGURE 1:**
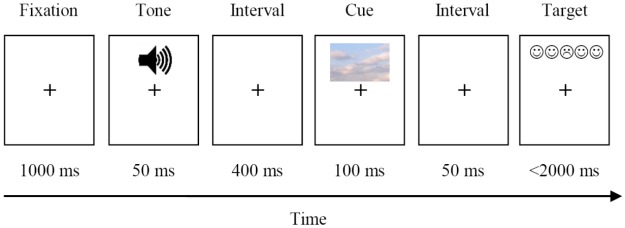
Experimental procedure of E-ANTI.

Each trial started with the presentation of a fixation plus sign for 1000 ms in the middle of the computer screen. In tone trials alerting signal was delivered for 50 ms. No alerting tone was presented in no-tone trials. Next, an asterisk was presented for 400 ms. After the asterisk disappeared, a cue was presented for 100 ms. The cue was presented horizontally centered, with the center of the cue being positioned 2.03 cm above or below the center of the screen with equal frequency. After a cuing interval of 50 ms, a target and distractors replaced the cue either at the same position (valid cue condition), or at the opposite position (invalid cue condition). The target remained on the screen for 2,000 ms or until the participants’ response. This sequence of events is depicted in **Figure 1**.

Apart from subjecting the data of the E-ANTI to full-factorial analyses of variance (cf. Results), several psychometric scores based on main effects were derived from these data. Alerting efficiency was defined by subtracting reaction time (RT) of tone trials from RT of no tone trials (i.e., alerting efficiency). Larger numbers of difference between tone and no tone trials indicate that participants benefit more from tone trials reflecting more efficient performance. Orienting efficiency was measured by subtracting RT of trials with the valid cue from those with the invalid cue (i.e., orienting efficiency). Larger differences of RTs are assumed to arise because of a difficulty to relocate attention after an invalid cue was presented. The measure for Executive efficiency was the difference between RT of target congruent trials and RT of target incongruent trials (i.e., executive efficiency). The greater the difference, the greater the difficulty to resolve cognitive conflict which reflects less efficient performance. In sum, in contrast to Alerting, higher scores represent less efficient processing in case of Orienting (i.e., less efficient reorienting after invalid cues) and Executive efficiency (i.e., more distraction by incongruent distracters).

## Results

### Subjective Measures

All statistical analyses were conducted using IBM SPSS Statistics 25.0. The raw data underlying the analyses can be found in **Supplementary Datasheet 1**. Independent t-tests showed that prior to stress induction, stress levels in the high-stress and the low-stress group were nearly equal (high-stress: *M* = 3.67, *SD* = 4.02; low-stress: *M* = 4.08, *SD* = 3.90). After stress induction, participants of the high-stress group reported significantly higher stress levels (*M* = 9.63, *SD* = 3.88) than participants of the low-stress group (*M* = 6.78, *SD* = 4.73; *t* = -2.58, *p* = 0.013). As paired t-tests demonstrated, stress levels significantly increased in both high-stress (*t* = -7.88, *p* = 0.001) and low-stress groups (*t* = -3.31, *p* = 0.002).

Similarly, anxiety levels did not significantly differ between high-stress group (*M* = 36.90, *SD* = 1.46) and low-stress group (*M* = 37.48, *SD* = 1.20) prior to stress induction. After stress induction, anxiety was marginally higher in the high-stress group (*M* = 45.17, *SD* = 2.06) than in the low-stress group (*M* = 41.45, *SD* = 1.64). These differences were non-significant (*t* = -1.41, *p* = 0.16). Anxiety levels significantly increased in both high-stress (*t* = -5.64, *p* = 0.001) and low-stress groups (*t* = -2.56, *p* = 0.016).

### Acute Stress and Attention

We subjected mean individual reaction times (RTs) and error rates (ERs) into 2 × 2 × 2 × 2 × 2 × 2 × 2 analyses of variance (ANOVAs) with the between-subjects factor Condition (high vs. low stress) and the within-subjects factors Tone (tone or no-tone), Cue Valence (positive or negative), Cue Validity (valid or invalid), Target Congruity (congruent or incongruent), Target Valence (positive or negative), and Cue-Target Congruity (congruent or incongruent). Trials from the very first block were regarded as practice trials and excluded from the analyses. Error trials (2.9%) and trials which were preceded by error trials were also excluded. Significant interactions were followed by least significant difference (LSD) *post hoc* tests to explore which pairs of cell means are significantly different. **Table 1** contains the mean RTs and ERs for each factor condition in the high- and low-stress group.

**Table 1:** Mean reaction times and proportions of error rates for each experimental condition.

	Alerting cue condition		Orienting cue condition		Orienting cue valence		Target valence		Cue-target valence congruity	
Group and congruity condition	*No tone*	*Tone*	*Valid*	*Invalid*	*Negative*	*Positive*	*Negative*	*Positive*	*Incongruent*	*Congruent*
*Reaction times*										
Low-stress										
Incongruent	766 (144)	765 (138)	765 (142)	766 (139)	768 (146)	762 (136)	766 (141)	764 (140)	761 (141)	769 (141)
Congruent	721 (137)	718 (134)	710 (135)	729 (136)	725 (140)	714 (132)	727 (138)	713 (133)	722 (136)	718 (136)
High-stress										
Incongruent	735 (133)	736 (125)	729 (129)	742 (129)	735 (130)	735 (128)	726 (125)	745 (131)	733 (129)	737 (129)
Congruent	694 (124)	689 (121)	681 (121)	702 (123)	694 (122)	690 (123)	694 (121)	690 (122)	693 (125)	690 (121)
*Error rates*										
Low-stress										
Incongruent	2.88 (0.15)	3.55 (0.18)	3.90 (0.18)	2.55 (0.14)	2.62 (0.15)	3.81 (0.18)	2.96 (0.15)	3.48 (0.17)	3.03 (0.15)	3.42 (0.17)
Congruent	1.60 (0.11)	1.50 (0.11)	1.40 (0.10)	1.70 (0.11)	1.59 (0.11)	1.51 (0.11)	1.65 (0.11)	1.44 (0.10)	1.74 (0.12)	1.35 (0.10)
High-stress										
Incongruent	4.91 (0.20)	4.12 (0.18)	5.31 (0.21)	3.72 (0.17)	4.12 (0.18)	4.91 (0.20)	3.07 (0.16)	5.96 (0.21)	4.16 (0.18)	4.87 (0.20)
Congruent	1.78 (0.12)	1.76 (0.12)	1.91 (0.12)	1.64 (0.11)	2.08 (0.13)	1.49 (0.11)	1.83 (0.11)	1.72 (0.12)	1.65 (0.11)	1.90 (0.12)


*Reaction times (RTs) are in milliseconds. Error rates are in proportions. SDs are given in parentheses.*

In the analysis of RTs, significant main effects of Cue Validity [*F*(1, 59) = 40.01, *p* = 0.001, partial η^2^ = 0.404], Target Congruity [*F*(1, 59) = 397.39, *p* = 0.001, partial η^2^ = 0.871], and Cue Valence [*F*(1, 59) = 15.44 *p* = 0.001, partial η^2^ = 0.207] were observed. Participants responded faster on validly (*M* = 721 ms, *SD* = 161) than on invalidly (*M* = 735 ms, *SD* = 162) cued trials. Congruent targets were associated with shorter RTs (*M* = 706 ms, *SD* = 158) than incongruent targets (*M* = 751 ms, *SD* = 161). Compared with positive cues (*M* = 726 ms, *SD* = 158), negative cues delayed participants’ responses (*M* = 731 ms, *SD* = 164).

In the following, we will restrict the report of significant interactions to those involving the between-subjects factor Condition. The analysis yielded a significant three-way interaction of Cue Validity, Target Congruity, and Condition [*F*(1, 59) = 4.42, *p* = 0.040, partial η^2^ = 0.070]. *Post hoc* analyses demonstrated faster RTs in trials with valid cues in both incongruent (728 vs. 742 ms, *p* = 0.001) and congruent conditions (681 vs. 702 ms, *p* = 0.001) in the high-stress group. In the low-stress group valid cues facilitated RTs only in congruent condition (congruent: 709 vs. 729 ms, *p* = 0.001, incongruent: 764 vs. 765 ms, n.s.).

Cue Valence interacted with Condition [*F*(1, 59) = 7.01 *p* = 0.01, partial η^2^ = 0.106]. *Post hoc* analyses revealed that compared to positive cues (*M* = 738 ms), negative cues significantly delayed RTs in the low-stress group (*M* = 746 ms, *p* = 0.001). In the high-stress group RTs in negatively cued trials (*M* = 712 ms) did not significantly differ from those in positively cued trials (*M* = 714 ms).

We also observed a significant interaction of Target Valence and Condition [*F*(1, 59) = 4.098, *p* = 0.047, partial η^2^ = 0.065]. *Post hoc* analyses indicated that RTs in negative target trials in the high-stress group (*M* = 710 ms) were marginally (*p* = 0.08) faster than in the low-stress group (*M* = 746 ms). The difference was non-significant in positive target trials (717 vs. 738 ms, n.s.). The effect of Target Valence was further qualified by a tendency toward a significant three-way Target Valence-by-Target Congruity-by-Condition interaction [*F*(1, 59) = 3.97 *p* = 0.052, partial η^2^ = 0.063]. Contrasting groups showed that in the low-stress group Target Valence significantly affected reaction times only in congruent condition. Compared to positive targets, negative targets prolonged reaction times in the low-stress group (712 vs. 726, *p* = 0.013). The opposite was true for high-stress group in which Target Valence only affected reaction times in incongruent condition, leading to faster reaction times in negative target trials than in positive target trials (726 vs. 744, *p* = 0.002). In both groups RTs were significantly faster in congruent condition than in incongruent condition (all *p*’s = 0.001).

Finally, we observed a significant three-way Cue-Target Congruity-by-Target Congruity-by-Condition interaction [*F*(1, 59) = 7.02, *p* = 0.010, partial η^2^ = 0.106]. The low-stress group was faster in positive target trials in the valence congruent condition (735 vs. 751, *p* = 0.003), but not in the incongruent condition (741 vs. 741, n.s.). Differences in RTs in the high-stress group were non-significant (congruent: 711 vs. 716, n.s.; incongruent: 709 vs. 717, n.s.). In the low-stress group, RTs differed significantly between congruent and incongruent conditions for both positive (735 vs. 741, *p* = 0.015) and negative target trials (751 vs. 741, *p* = 0.001).

The corresponding analyses of ERs revealed an almost identical pattern of results. For each main effect and interaction we found for RTs, we scrutinized the data for eventual speed-accuracy trade-offs. We observed a speed-accuracy trade-off only for the main effect of Cue Validity in that faster RTs in valid trials went along with increased ERs [*F*(1,59) = 23.88, *p* = 0.001, partial η^2^ = 2.88; valid: 0.032 vs. invalid: 0.025, *p* = 0.001]. To address this issue, we calculated the linear integrated speed-accuracy score (LISAS) as recommended by Vandierendonck (2017). The comparison of LISAS-scores between valid and invalid scores by t-test indicated that RTs corrected for the amount of incorrect responses were still significantly faster in valid than in invalid trials (*t* = -3.89, *p* = 0.001; *M* = 710835, *SD* = 407410 vs. *M* = 858705, *SD* = 519021). This means that the effect of Cue Validity was only partially attributable to speed-accuracy trade-off.

For the ERs, we will only report effects of Condition which go beyond those reported in the analysis of RTs. The analysis yielded a significant interaction of Tone and Condition [*F*(1,59) = 5.74, *p* = 0.02, partial η^2^ = 0.089]. *Post hoc* analyses indicated that ERs in no-tone trials in the high-stress group (*M* = 0.034) were marginally (*p* = 0.05) higher than in the low-stress group (*M* = 0.023). The difference was non-significant in tone trials (0.030 vs. 0.026, n.s.). There was a tendency toward a significant decrease of ERs as a function of tone presentation in the high-stress group (0.034 vs. 0.030, *p* = 0.053), whereas no such trend was observed in the low-stress group (0.023 vs. 0.026, n.s.).

We also observed a significant three-way Tone-by-Target Congruity-by-Condition interaction [*F*(1,59) = 6.99, *p* = 0.01, partial η^2^ = 0.106]. *Post hoc* tests demonstrated significantly higher ERs in the target-incongruent condition as compared to the target-congruent condition in both groups in both no-tone and tone trials (all *p*’s < 0.05). In the no-tone incongruent conditions, ERs were higher in the high-stress group compared to the low-stress group (*M* = 0.050 vs. *M* = 0.030, *p* = 0.021). Further, the low-stress group showed a significant difference of ER in no-tone trials between congruent and incongruent conditions, with larger RTs in congruent condition (0.030 vs. 0.037, *p* = 0.03). No such increase was observed in tone trials (0.016 vs. 0.015, n.s.). In contrast, ERs of the high-stress group were significantly larger in no-tone trials in incongruent condition compared to congruent condition (0.50 vs. 0.04, *p* = 0.012), whereas no change was observed in tone trials (0.019 vs. 0.018, n.s.).

### Subjective Stress Measures and Attention

In order to explore if subjective stress was related to the efficiency of any of the attention networks, we conducted additional correlation analysis separately for the high- and the low-stress group. Separate analyses for the two groups were based on the rationale that different levels of stress may be associated with different functional relationships between subjective stress and attention. Efficiency measures of each of the networks were correlated with changes of subjective stress levels (stress levels after stress induction or low-stress activity minus stress levels at baseline). The same was done with the state anxiety measures. No significant correlations were observed in the low-stress group (all *p*’s > 0.399). In the high-stress group, the Alerting efficiency negatively correlated with an increase in anxiety (*r* = -0.43, *p* = 0.018), indicating poorer efficiency of alerting with increasing anxiety. Further, we observed a positive association between Executive efficiency (inverted scale) and increases in state anxiety, meaning that higher increases were related to poorer Executive efficiency (*r* = 0.38, *p* = 0.036). Similarly, higher increases in stress level were also positively related to the Executive efficiency (inverted) score, with this correlation being marginally significant (*r* = 0.35, *p* = 0.06).

### Acute Stress and Cytokines

Prior to analyses the normality assumption was checked for all continuous variables. Cytokine data showed left skewed distributions requiring log-transformation. We assessed several control variables thought to be associated with cytokine levels, and hence might provide alternative explanations for any observed relationships between stress, attention and cytokine levels. These were age, sex, and body mass index (weight/height^2^). Each control variable was considered as a covariate. To check for randomization, low-stress and high-stress group were compared on control variables and baseline cytokine levels using t-tests (all *p*’s > 0.05).

To test if cytokine levels increased with increasing levels of stress and if attention would moderate the relationships between stress and cytokine responses, we conducted a series of linear mixed models with repeated measures. Each mixed model included fixed effects of Time (5 min post-stress, 45 min. post-stress), Condition (low-stress, high-stress), the efficiency scores of the particular attention network (Alerting, Orienting, and Executive efficiency as continuous predictor), their respective two-way interaction terms (Time-by-Condition, Time-by-network efficiency, Condition-by-network efficiency), and the three-way interaction term (Time-by-Condition-by-network efficiency). The predictor variables were calculated as described in section E-ANTI with higher scores reflecting more efficient Alerting and less efficient Orienting and Executive control (cf. E-ANTI). Age, sex, body mass index were entered into the model as fixed factors. To control for baseline imbalance, each post-stress cytokine score was adjusted for its baseline score by including it as an additional control variable in each model. This procedure was chosen due to its higher efficiency gains (e.g., power) in randomized controlled studies, as compared to the analyses of change scores (Vickers and Altman, 2001; van Breukelen and van Dijk, 2007). In order to account for heterogeneity across individuals in baseline cytokine levels and cytokine responses over time, subject was included as a random factor.

Each cytokine type was analyzed as a dependent variable. ANCOVA and regression models assume uncorrelated residuals. Since we used repeated measures design, multiple observations on cytokines originated from the same individuals. In this case, residuals from measurements next to each other might be correlated. For this reason, we set covariance structure type to autoregressive (AR1). For convenience, continuous explanatory variables were centered (i.e., by subtracting the respective sample mean) prior to analyses. Categorical explanatory variables (Condition, Time) were dummy-coded. Interaction effects were tested by simple effects tests which were adjusted for multiple comparisons (Bonferroni). Pairwise comparisons were based on estimated marginal means (EM-means, adjusted for age, sex, and body mass index). In order to interpret significant interactions including attention network efficiency variables, we divided the sample by median split into participants with low and high efficiency of each of the attention networks.

Since each cytokine type was treated as a separate dependent variable, the analyses produced multiple hypothesis tests. Sequential Bonferroni-Holm procedure was used to control for the family-wise error rate and thus reduce the probability of Type I error (Holm, 1979). This method proved to be statistically more powerful than one-step Bonferroni correction method (Aikin and Gensler, 1996). Due to the exploratory character of probing the interrelations between cytokine levels and attention measures, correction for multiple comparisons was applied only to *post hoc* tests.

**FIGURE 2:**
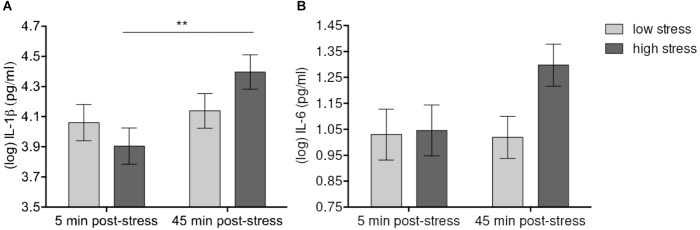
Effects of acute laboratory stress level on IL-1β **(A)** and IL-6 **(B)**. Values represent (log-transformed) mean saliva levels of IL-1β and IL-6 adjusted for baseline measure, age, sex, and body mass index in samples obtained 5 and 45 min after highly stressful vs. lowly stressful task. Error bars show standard errors +/-1 (SE). IL-1β significantly increased and at 45 min. post-stress in the high-stress group. The corresponding increase of IL-6 failed to reach significance after correction for multiple comparisons. ^∗∗^*p* ≤ 0.01.

Levels of psychological stress had differential effects on IL-1β and IL-6. Mixed model analyses showed a significant effect of Time [*F*(1,56) = 8.43, *p* = 0.006] and a Time-by-Condition interaction effect [*F*(1,56) = 5.06, *p* = 0.028 > *p*_adj_ = 0.01] on levels of IL-1β. IL-1β increased up to 45 min. after stress manipulation in both high- and low-stress groups. Although after correction for multiple comparisons, the Time-by-Condition interaction failed to reach significance, the increase of IL-1β was still significant in the high-stress group (*p* = 0.004) but not in the low-stress group (n.s.). Similarly, there was a trend toward a significant sample Time-by-Condition interaction effect on IL-6 [*F*(1, 56) = 2.42, *p* = 0.12], demonstrating an increase of IL-6 levels up to 45 min. after stress manipulation in the high-stress group (*p* = 0.168 vs. *p*_raw_ = 0.048), but not in the low-stress group (n.s.) (**Figure 2**). No further statistically significant effects of Condition (all *p*’s > 0.23) or the Condition-by-Time interaction (all *p*’s > 0.11) were detected, demonstrating that stress levels did not affect cytokine increases in different ways.

Repeated measures mixed models showed instead that both high and low stress led to an increase of cytokines. As **Table 2** demonstrates, levels of IFN-α, IFN-γ, TNF-α, IL-5, and IL-10 significantly increased 45 min. after stress induction.

**Table 2:** Descriptive statistics of cytokine concentrations in samples obtained 5 and 45 min after stress induction.

Cytokine type	5 min	45 min			
	Mean (SE)	Mean (SE)	F (df1, df2)^a^	p	p_adj_
IFN-α	1.43 (0.16)	1.82 (0.18)	13.07 (1,56)	**0.001**	**0.004**
IFN-γ	5.25 (0.82)	6.83 (0.81)	11.74 (1,56)	**0.001**	**0.004**
TNF-α	1.95 (0.19)	2.7 (0.29)	12.34 (1,56)	**0.001**	**0.005**
IL-2	27.1 (4.32)	37.8 (5.56)	4.59 (1,56)	0.036	0.01
IL-4	43.2 (5.96)	53.6 (8.01)	1.86 (1,56)	0.178	n.s.
IL-5	18.5 (3.23)	25.6 (4.14)	9.84 (1,56)	**0.003**	**0.005**
IL-8	1914.4 (164.92)	2254.42 (230.54)	5.63 (1,56)	0.02	0.01
IL-10	6.5 (1.01)	9.7 (1.5)	16.78 (1,56)	**0.001**	**0.003**
IL-12p70	4.9 (0.95)	6.06 (1.02)	4.32 (1,56)	0.04	0.01
IL-13	13.3 (1.71)	17.1 (2.07)	6.55 (1,56)	0.013	0.008
IL-17A	55.7 (10.25)	60.4 (10.85)	2.28 (1,56)	0.136	n.s.


*Values represent (row) means. Standard errors (SE) are shown in parentheses. ^a^Repeated measures linear mixed models analyses were used to compare mean cytokine concentrations 5 and 45 min after completion of stressful task.*

### Gender Differences

The analyses revealed a significant effect of gender on IL-8 [*F*(1, 53) = 7.2, *p* = 0.01]. Levels of IL-8 were significantly higher in men than in women (*p* = 0.01). Trends toward effects of gender were also detected for IL-1β and IL-6 (both *p*’s > 0.07) with cytokine levels being higher in men than in women. No significant Gender-by-Time, Gender-by-Condition, and Gender-by-Time-by-Condition interactions were observed, indicating that levels and increases of cytokines were nearly equal for men and women in both groups.

### Acute Stress, Attention, and Cytokines

**FIGURE 3:**
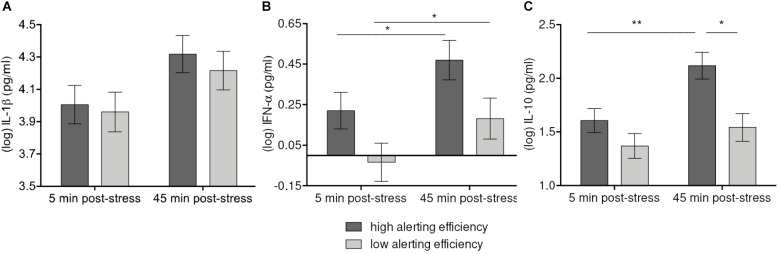
Moderation of IL-β **(A)**, IFN-α **(B)**, and IL-10 **(C)** increases 45 min after stress induction by Alerting efficiency. Values represent (log-transformed) means adjusted for baseline measure, age, sex, and body mass index. Error bars show standard errors +/-1 (SE). Alerting efficiency scale was divided at the median into high and low Alerting efficiency. High Alerting efficiency was associated with greater stress-induced responses of IFN-α, and IL-10 in both high-stress and low-stress groups. The increase of IL-β failed to reach significance after correction for multiple comparisons. ^∗^*p* ≤ 0.05, ^∗∗^*p* ≤ 0.01.

Mixed linear models analyses revealed a significant sample Time-by-Alerting network efficiency effect on IL-1β [*F*(1, 52) = 4.08, *p* = 0.048] (**Figure 3A**). The increase of IL-1β up to 45 min. after stress induction was higher with high Alerting efficiency (*p* = 0.084 vs. *p*_raw_ = 0.021) than with low Alerting efficiency (n.s.). Although only marginally significant after Bonferroni correction, the observed trend indicated a more pronounced IL-1β response if Alerting efficiency was high.

Similarly, Alerting efficiency was associated with IFN-α levels and their stress-induced changes [*F*(1, 52) = 3.99, *p* = 0.051]. On the descriptive level, concentrations of IFN-α were higher both at 5 min. and 45 min. post-stress with high Alerting efficiency. Compared to low Alerting efficiency (*p* = 0.048), high Alerting efficiency was related to a greater increase of IFN-α levels (*p* = 0.028) (**Figure 3B**). Further, we observed a significant Time-by-Alerting efficiency effect on IL-10 [*F*(1, 52) = 6.63, *p* = 0.013]. IL-10 showed a greater increase (*p* = 0.004) and higher levels at 45 min. post-stress (*p* = 0.012) with high efficiency than with low efficiency (n.s.) (**Figure 3C**). In sum, higher Alerting efficiency was significantly related to higher levels of IL-10 after stress exposure and greater increase of IL-10 and IFN-α.

**FIGURE 4:**
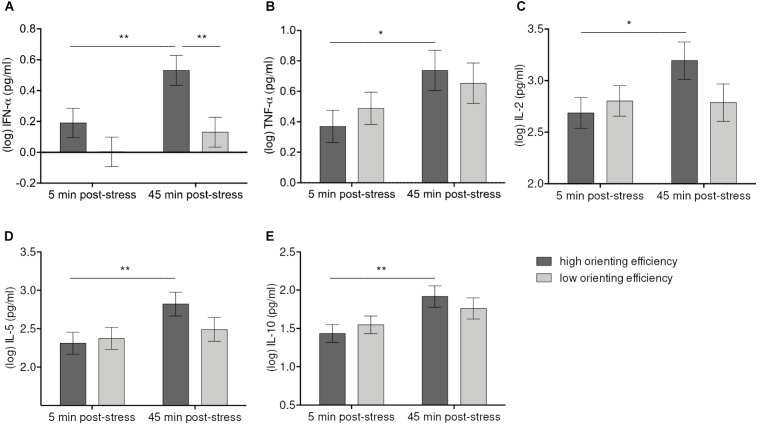
Moderation of IFN-α **(A)**, TNF-α **(B)**, IL-2 **(C)**, IL-5 **(D)**, IL-10 **(E)** increases 45 min after stress induction by Orienting efficiency. Values represent (log-transformed) means adjusted for baseline measure, age, sex, and body mass index. Error bars show standard errors +/-1 (SE). Orienting efficiency scale was divided at the median into high and low Orienting efficiency [values below the median were considered as high Orienting efficiency because the scale is inverted (cf. E-ANTI)]. High Orienting efficiency was associated with greater responses of IFN-α, TNF-α, IL-2, IL-5, IL-10 45 min. post-tress in both high-stress and low-stress groups. ^∗^*p* ≤ 0.05, ^∗∗^*p* ≤ 0.01.

The Time-by-Orienting interaction was statistically significant for IFN-α [*F*(1, 56) = 5.56, *p* = 0.021], TNF-α [*F*(1, 56) = 7.92, *p* = 007], IL-2 [*F*(1, 56) = 7.11, *p* = 0.010], IL-5 [*F*(1, 55.9) = 7.14, *p* = 0.010], and IL-10 [*F*(1, 56) = 4.59, *p* = 0.036] (**Figure 4**). Simple effects tests corrected for multiple comparisons showed that high Orienting efficiency was associated with more pronounced cytokine responses. Thus, participants better in Orienting efficiency showed significant increases of IFN-α (*p* = 0.004), TNF-α (*p* = 0.04), IL-2 (*p* = 0.012), IL-5 (*p* = 0.004), and IL-10 (*p* = 0.004) from 5 min. post-stressor to 45 min. post-stressor. The cytokine levels difference for high and low Orienting efficiency at 45 min. post-stressor was significant only for IFN-α (*p* = 0.008).

The analyses further revealed significant Condition-by-Orienting efficiency effects on IFN-α [*F*(1, 52) = 5.79, *p* = 0.02], IFN-γ [*F*(1, 50.42) = 18.06, *p* = 0.001], and IL-12p70 [*F*(1, 52) = 8.74, *p* = 0.005], demonstrating that relationships between orienting attention and cytokine levels varied with levels of stress. Bonferroni adjusted simple effects tests demonstrated that levels of IFN-γ (*p* = 0.036) and IL-12p70 (*p* = 0.044) were significantly higher with high Orienting efficiency than with low Orienting efficiency, but this applied only to the high-stress group. Levels of IFN-α were also higher with high Orienting efficiency in the high-stress group, but the difference was non-significant at adjusted p-value (*p* = 0.17 vs. *p*_raw_ = 0.043). No significant differences were observed in the low-stress group. Similarly, we found significant Condition-by-Orienting efficiency effects on IL-10 [*F*(1, 52) = 10.99, *p* = 0.002], IL-13 [*F*(1, 52) = 10.5, *p* = 0.002], IL-2 [*F*(1, 52) = 10.87, *p* = 0.002], and IL-4 [*F*(1, 52) = 7.98, *p* = 0.007]. Although simple effects tests revealed no significant differences, there was a trend toward higher cytokine levels in the low-stress group as compared to high-stress group if Orienting efficiency was low (IL-10: *p* = 0.11, IL-13: *p* = 0.07, IL-2: *p* = 0.09, IL-4: *p* = 0.15). Thus, in high-stress condition levels of IFN-α, IFN-γ, and IL-12p70 were positively associated with high Orienting efficiency, whereas in low-stress condition levels of IL-2, Il-4, IL-10, and IL-13 were higher if Orienting efficiency was low.

Finally, we observed a significant three-way interaction effect of Time-by-Condition-by-Executive efficiency on IL-17A levels [*F*(1, 56) = 5.12, *p* = 0.027]. Bonferroni adjusted *post hoc* tests showed a trend toward a negative association between Executive Efficiency and IL-17A in the low-stress group. However, the differences failed to reach a significance level after correction for multiple comparisons. Compared to high-stress group, higher efficiency in the low-stress group was associated with lower IL-17A both at 5 min. post-stress (*p* = 0.152 vs. p_raw_ = 0.019) and 45 min. post-stress (*p* = 0.64 vs. p_raw_ = 0.08).

No further significant Time-by-Condition-by-attention network efficiency interaction effects on cytokine reactivity were observed, indicating that modulating effects of attention processes on cytokine reactivity over time were similar for both high-stress and low-stress groups.

### Additional Analyses

So far, the attention network measures were collapsed across positive and negative stimuli. In order to tap eventual valence-specific effects, we tested modulating effects of stress and attention on cytokine reactivity depending on stimulus valence (positive or negative). To this end, we first calculated change scores (second post-stress sample – first post-stress sample) for each cytokine type. We further calculated Orienting and Executive network efficiency separately for positive and negative stimuli. Positive Orienting efficiency was calculated by subtracting RTs in trials with positive valid cues from RTs in trials with positive invalid cues. Negative Orienting efficiency was calculated in the same way using negatively cued trials. For Executive network, efficiency was computed by subtracting congruent from incongruent trials separately for positive and negative targets. We applied partial correlation analyses to test for relationships between cytokine changes and attention networks efficiency for emotional stimuli. Age, sex, and body mass index were included into analyses as control variables.

The analyses revealed negative correlations between negative Orienting efficiency scores and changes of IFN-α (*r* = -0.28, *p* = 0.042), TNF-α (*r* = -0.33, *p* = 0.016), IL-2 (*r* = -0.43, *p* = 0.001), IL-10 (*r* = -0.37, *p* = 0.007) IL-4 (*r* = -0.34, *p* = 0.012, IL-5 (*r* = -0.40, *p* = 0.002), and IL-13 (*r* = -0.45, *p* = 0.001). Since higher scores reflect poorer performance (cf. E-ANTI), these relationships indicate that high Orienting efficiency in negatively cued trials is linked to greater increases of cytokines. No systematic relationships were observed for cytokines and positive Orienting network efficiency. To examine if condition would alter the relationships, we ran the correlation analyses separately for low-stress and high-stress group. The pattern of associations between negative Orienting network efficiency did not substantially differ between the low-stress and high-stress groups.

**FIGURE 5:**
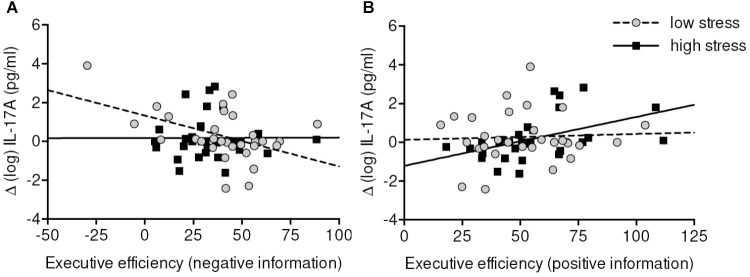
Correlations between (emotional) Executive efficiency and change scores of IL-17A in low-stress **(A)** and high-stress group **(B)**. Executive efficiency scale is inversely scored (=lower values indicate higher efficiency). Data were analyzed by partial correlation analyses controlling for baseline measure, age, sex, and body mass index. Higher Executive efficiency in processing negative information was associated with greater increases of IL-17A in the low-stress group. Higher Executive efficiency in processing positive information was associated with lower increases of IL-17A in the high-stress group. It has to be noted that when the very left data point **(A)** is removed from the analyses the correlation drops down to *r* = –0.32 (*p* = 0.11).

For emotional Executive network efficiency, we detected a mutual dissociation between low-stress and high-stress group and positive and negative stimuli. Higher increases of IL-17A in the low-stress group were negatively correlated with (inversely scored) negative Executive network efficiency (*r* = -0.43, *p* = 0.034), whereas in the high-stress group increases of IL-17A were positively correlated with (inversely scored) positive Executive network efficiency (*r* = 0.50, *p* = 0.014). In other words, higher efficiency in processing negative information was related to increases of IL-17A in the low-stress group. In the high-stress group higher efficiency in processing positive information was related to lower increases of IL-17A (**Figure 5**).

### Subjective Stress Measures and Changes in Cytokine Levels

In order to test for associations between stress and stress-induced cytokine changes, we conducted additional correlation analysis separately for the high- and low-stress groups. To this end, we first calculated cytokine change scores by subtracting sample 2 (5 min. post-stress) from sample 3 (45 min. post-stress). We correlated cytokine change scores of each cytokine type with changes of subjective stress levels (stress levels after stress induction minus stress levels at baseline). The same was done with the state anxiety measures. We observed positive correlation between subjective stress levels and levels of INF-α (*r* = 0.46, *p* = 0.01) and IL-12p70 (*r* = 0.48, *p* = 0.001) in the low-stress group. No relationship between subjective stress measures and changes in cytokine levels were detected in the high-stress group. Correlation analyses using the whole sample revealed no significant association either.

## Discussion

The purpose of our research was to examine the impact of acute mental stress on levels of salivary cytokines and different dimensions of attention to emotional information (alerting, orienting, and executive network) measured by emotional attention network test integration (E-ANTI). Furthermore, we examined if efficiency of each of the attention networks would be associated with cytokine changes induced by psychological stress. Cytokines were determined from saliva with saliva samples having been collected before, 5 min after, and 45 min after stress exposure. The stress protocol applied in our study was designed to induce different levels of mental stress, high and low stress. The high-stress condition consisted of a socially evaluated PASAT. In the low-stress condition a paper-and-pencil arithmetic task without socially evaluation was used.

The data showed greater increase of IL-1β and a trend toward a greater increase of IL-6 in high-stress condition. Increases of further cytokines did not vary with varying levels of stress. Instead, IFN-α, IFN-γ, TNF-α, and IL-10 increased in both conditions. The second question was if levels of stress would predict efficiency of emotional attention networks. The main results were interactions between experimental condition and emotional valence of the stimuli. These interactions indicated that participants who were exposed to PASAT reacted faster to negative stimuli. In contrast, participants who underwent low-stress activity showed improved performance in positive cue and positive target trials. The third hypothesis predicted that emotional network attention efficiency would be related to cytokine responses. Higher Alerting Efficiency was positively associated with higher increases in IFN-α and IL-10. Higher Orienting efficiency was positively associated with higher increases in IFN- α, TNF- α, IL-2, IL-5, and IL-10.

At the outset, we point out that our study is limited by the lack of a basal rest condition, which was not possible to obtain within the parameters of our study design, and our analysis and discussion of the results obtained, below, reflects this limitation. Thus, apart from distinctive effects of the stress manipulation on IL-1β, IL-6 and stimulus valence in the attention task, contrasting cytokine and attention measures between the high- and low-stress groups failed to yield significant effects of stress intensity. The low-stress condition may be an inappropriate reference measure to establish potential effects of stress, given potentially stressful features of the experimental situation, therefore decreasing differences in stress experience between the high- and the low-stress group. Second, the speeded attention task which was carried out before the last saliva collection might have contributed to increased stress levels in both conditions. Timing of the sampling is another critical variable that may require further attention in future studies. Nevertheless, the data as obtained offer significant insights into the association of cytokine responses to psychological stress with attention to emotional information, as discussed in detail below.

### Acute Stress and Attention

Apart from an impact of stress on error rates regarding Alerting network efficiency, there were no overall effects of condition on any of the attention networks. However, emotional stimulus valence had differential effect on participants’ performance depending on stress level. Participants in the high-stress condition reacted faster after the presentation of a negative cue. In the same vein, negative targets also improved performance in this group. In contrast, the low-stress group took longer to respond when they were faced with negative cues or targets. In other words, negative information disrupted information processing in the low-stress group and facilitated performance in the high-stress group. The interference of negative cues with performance in the low-stress group may reflect a relatively automatic increase of the salience of negative stimuli that enhances their processing at the cost of task-related cognitive processes. As research demonstrates, negative stimuli have a greater disruptive effect on other cognitive processes than positive stimuli (Pratto and John, 1991; Williams et al., 1996; Wentura et al., 2000). Faster reaction times in negative cue or target trials in high-stress group are in agreement with the mood congruence hypothesis (Colombel, 2007). This hypothesis implies that individuals in a positive emotional state preferentially process positive information, and individuals in a negative emotional state favor negative information (Bower, 1981). In our study, high stress might have had mood-congruent effects on attention increasing the selectivity to negative information which is in line with previous research (Mogg et al., 1990; Richards and Christopher, 1992; Becker and Leinenger, 2011).

### Acute Stress and Cytokines

Our results demonstrated that increases in IL1-β and IL-6 varied with levels of stress (see **Figure 1**). In the high-stress condition, IL-1β significantly increased at 45 min. following socially evaluative PASAT. The IL-6 response was also observed to be more pronounced in the high-stress condition, however, the increase failed to reach significance after correction for multiple comparisons. Although the observed effects of condition are quite small, this finding corroborates previous results on these cytokines measured in blood and saliva (Steptoe et al., 2007; Slavish et al., 2015). Regarding further cytokines, IFN-α, IFN-γ, TNF-α, and IL-10 showed significant increases in both groups 45 min. after the stress task (see **Table 2**). Thus, for these cytokines our stress manipulation failed to yield a clear-cut effect of stress intensity. There are several reasons why levels of cytokines increased in both conditions. First, socially evaluative mental stress in the high-stress condition might have not been powerful enough to induce greater cytokine response than the low-stress task. Second, the stress level induced by the arithmetic task in the low-stress condition might have been too high resulting in ceiling effects for IFN-α, IFN-γ, TNF-α, and IL-10 already at this level of stress. Third, further design issues, such as the laboratory setting itself or the speeded nature of the attention measurement before the last saliva measurement might have increased stress in both conditions to the same degree. However, this explanation is not entirely supported by the data. Although subjective stress and anxiety increased in both groups, subjective stress was significantly higher in the high-stress group. In addition, if stress levels were too similar in the high- and low-stress groups, this would be presumably also reflected in IL-1β and IL-6. It is conceivable that timing of the sampling was one of the reasons for the lack of differences in cytokine responses between conditions. The first saliva sample was taken almost immediately after arrival at the laboratory. It is not possible to rule out that saliva collection along with the novelty of the laboratory situation contributed to increased stress in all participants. Furthermore, the observation period may have been too short for some treatment effects to be detected. In blood many inflammatory biomarkers peak between 60 and 120 min post-stressor (Steptoe et al., 2007). A few recent studies showed that salivary IL-1β and IL-6 increased already after 10 min after acute stress exposure, indicating that at least some salivary cytokines rise earlier than those in blood (e.g., Mastrolonardo et al., 2007). However, temporal details for salivary assessments of reactivity of IFN-α, IFN-γ, and IL-10 are less conclusive (Slavish et al., 2015). It seems that stress sensitivity is most reliable for IL-1β and IL-6, and our data support this.

### Acute Stress, Attention, and Cytokines

With regard to the primary concern of our study, we found evidence that attention processes modulated the impact of stress on cytokine responses. Increases in cytokines after stressful task varied depending on the efficiency of attention networks. High efficiency of the Alerting network was associated with stronger responses of IL-1β, IFN-α, and IL-10. In both the low-stress and the high-stress group, increases of cytokines were more pronounced in participants with higher Alerting efficiency (see **Figures 3A–C**). This result is consistent with a small number of studies linking norepinephrine, which is the main neurotransmitter controlling the alerting system (Green et al., 2008), acute stress, and cytokines (van der Poll et al., 1994; Maes et al., 2000). Some evidence indicates that catecholamines stimulate pro-inflammatory response via adrenergic receptor binding (Bierhaus et al., 2003; Wolf et al., 2009). Further research is needed to understand the mechanisms underlying the relationship between Alerting network efficiency and cytokine stress response.

Higher efficiency of the Orienting network was furthermore related to greater elevations of IFN-α, TNF-α, IL-2, IL-5, and IL-10 (see **Figures 4A–E**). Importantly, this interaction was based on Orienting efficiency only in negatively cued trials, and not in positively cued trials. In addition, the overall levels of IFN-α, IFN-γ, and IL-12p70 were higher with high Orienting efficiency in the high-stress group. Faster reaction times after the presentation of a negative cue and concomitant cytokine elevations in both groups with higher cytokine levels in the high-stress group may represent an attempt to counteract the impact of potentially harmful stimulation, thereby down-regulating the affective and physiological stress reactions. Although no studies have investigated the association between orienting attention and cytokine reactivity, prior studies on attentional bias and cortisol demonstrated that attention away from negative information was associated with blunted cortisol increase in response to stress and negative stimuli (van Honk et al., 2000; Ellenbogen et al., 2002). In sum, there seems to be a bias toward or away from negative information in the high-stress group which is associated with greater cytokine responses. In contrast, in the low-stress group we observed the opposite association in that cytokine levels were higher in participants with low Orienting efficiency.

Contrary to our expectation, we observed little evidence for the relationship between executive control and cytokines. On the descriptive level, higher efficiency of the Executive network was associated with lower levels of IL-17A in the low-stress group. No relationship was detected in the high-stress group, suggesting that the modulating effects of Executive efficiency take place only if stress is low. To our knowledge, no study has linked IL-17A to psychological stress and Executive attention before. There is one study which examined the associations between cognitive control and pro-inflammatory cytokines. Specifically, Shields et al. (2016) used an emotionally evocative video to induce stress in a randomized design. Afterward, participants’ executive attention of emotional information was measured by an emotional Stroop task (Shields et al., 2016). Levels of salivary IL-1β, IL-6, and IL-8 were assessed at baseline and after stress induction or control activity. These researchers could show that better cognitive control of emotional information in the stress group was associated with less pronounced pro-inflammatory cytokines responses following the emotional video. This interaction was not detected in the control group. Additionally, in our study, reactivity of IL-17A was co-influenced by target valence. Greater increases of IL-17A were associated with lower Executive network efficiency to negative information in the low-stress group (see **Figure 5A**) whereas the high-stress group exhibited greater increases when Executive network efficiency to positive information was low (see **Figure 5B**). This result is also in agreement with the study of Shields et al. (2016) showing that immune reactivity is not primarily linked to Executive control in a valence-independent manner, but also depends on the emotional valence of information.

Increases of pro- and anti-inflammatory cytokines observed in our study can be interpreted as a part of an adaptive stress response. Facing a stressful challenge, an organism prepares for a fight-or-flight reaction and its possible consequences (e.g., injury, infection). As mediators of inflammation, cytokines play a key role in wound healing and defending the body against infections. That is, higher levels of cytokines along with an activated HPA-Axis imply protective effects of acute stress (Dhabhar, 2014). As recent data shows, cytokines can directly and indirectly modulate cognition (Wilson et al., 2002). Specifically, cytokines induce sickness behavior which aims to combat the pathogens, while sustaining the ability to interrupt this energy-saving state in case of defensive responses to further environmental threats being necessary (Aubert, 1999). Although very tentative, the link between higher levels of cytokines and higher efficiency of alerting and orienting attention can be interpreted in terms of sickness behavior. Thus, the increase of cytokines might have boosted attention performance in order to monitor the environment for other potential threats.

However, potential modulation of cytokine responses through attention processes should also be considered. Shifting attention toward or away from certain aspects of a situation is one of the strategies to regulate emotions (Gross, 2001). According to this line of reasoning, more efficient processing of negative information may induce additional psychological stress leading to a higher increase in cytokines. Further studies are required to support this pathway.

Finally, psychological stress and cytokine responses may produce synergistic effects on attention by influencing cognition and behavior through functionally similar pathways. For example, in the study of Brydon et al. (2009), psychological and immunological stress (vaccination) jointly led to a higher increase in negative mood and IL-6 compared to conditions in which only psychological or only immunological stress was induced. Our data may be interpreted in a similar fashion. There were no differences between the high- and low-stress groups for Alerting and Orienting attention, as well as IFN-α, IFN-γ, TNF-α, and IL-10. However, combining both cognitive and immunological parameters yielded several interrelations between them, and in case of orienting attention a trend toward a stronger association in the high-stress group was observed. Thus, Orienting attention away from negative stimuli and increased cytokine responses may be part of the same overall response to stress that protects the organism from the impact of noxious stimuli both at a psychological and a immunological level.

### Limitations

Several limitations of our study have been noted earlier in the discussion. Another limitation of our study is that the first saliva sample was collected shortly after participants arrived at the laboratory. Thus, it cannot be ruled out that this first sampling was affected by the novelty of the laboratory setting, which could have contaminated the baseline measurement especially in the low-stress group. Some relaxation period before collecting the first sample or including a sample taken at home should be considered in future studies. Furthermore, the last saliva sample was collected 45 min. after completion of the stress task. Since levels of the most cytokines were elevated at that time of measurement, it is unclear whether the cytokine stress response already achieved its maximum at this time. Further research with additional samples taken at later time points after the stressor is needed. Second, there was no rest condition in our study. As indicated by the results, our stress manipulation failed to induce differences in responses of several cytokines between the high-stress and low-stress groups. Therefore, it may be beneficial to include a no-stress (i.e., rest) control condition to determine the associations of immune and cognitive parameters unaffected by stress. However, it should be noted that any laboratory procedure will induce some amount of stress in naïve participants, making it hard to realize a no-stress condition in a strict sense. One way to deal with this problem is to implement a habituation phase to make participants familiar with the laboratory setting and saliva sampling. Finally, our results on the relationships between cytokine responses and attention measures are correlational. Future studies should thus examine, if e.g., experimental stimulation of cytokine production would affect attention networks, or, vice versa, if manipulation of attention networks would have impact on cytokine reactivity.

### Summary and Implications

Taken together, higher intensity of acute laboratory mental stress induced an increase in IL-β and a marginal increase in IL-6. Levels of IFN-α, TNF-α, IFN-γ, and IL-10 increased in response to both high and low intensity of the stressor. Stress-induced cytokine responses were associated with the efficiency of attention networks. Furthermore, this relation partially depended on the valence of stimuli used in the attention task such that cytokine increases were closer related to attention to negative information than to positive information. The associations between acute stress, attention to emotional information, and cytokine responses are relevant for understanding individual differences in stress reactivity. In addition to studies examining the modulatory role of personality traits in immunity, research on cognitive processes such as attention promises to shed new light on the relationship between psychological and immunological factors, e.g., mechanisms underlying the link between stress and mental and physical health outcomes. To date, attention processes and stress-induced inflammation have been closely related to pathology of depression and anxiety disorders by two independent lines of research. Biased attention to emotional information plays a key role in stress reactivity and in the development and persistence of symptoms of affective disorders (Mogg and Bradley, 2005). In various models, depression and anxiety are reliably correlated with increased inflammation (Raison et al., 2006; Hou et al., 2017). With regard to the fact that the intensity of psychological and biological acute stress reactivity was proved to be predictive for emotional and physical consequences of stress, modifying stress-related emotions and cognitions might be a target for the development of interventions to prevent or treat affective disorders. Although the direction of the relationships between cytokine stress responses and attention processes is only beginning to be understood, recent studies confirm that better cognitive control measured under stress predicted attenuated links between participants’ recent life stress exposure and their current health complaints (Shields et al., 2017) and the severity of depression symptoms (Quinn and Joormann, 2015). More research is needed to understand the biological and neurocognitive pathways that underlie the interaction between attention processes and cytokine responses after stressful challenge, and how their interplay influences health.

## Data Availability Statement

All immunological and psychological data obtained and analyzed for the present study are available in the **Supplementary File**.

## Ethics Statement

This study was carried out in accordance with the recommendations of the local Ethics Committee of the Leibniz Research Centre for Working Environment and Human Factors. The protocol was approved by the local Ethics Committee of the Leibniz Research Centre for Working Environment and Human Factors. All subjects gave written informed consent in accordance with the Declaration of Helsinki.

## Author Contributions

VM and TK designed the study. MC and VM collected the data. All authors analyzed and interpreted the data. VM prepared the manuscript, which was revised by TK, MC, and CW. All authors agreed to be accountable for the content of the work.

## Conflict of Interest Statement

The authors declare that the research was conducted in the absence of any commercial or financial relationships that could be construed as a potential conflict of interest.
